# Abomasal infusion of branched-chain amino acids or branched-chain keto-acids alter lactation performance and liver triglycerides in fresh cows

**DOI:** 10.1186/s40104-023-00973-7

**Published:** 2024-01-29

**Authors:** Kristen Gallagher, Isabelle Bernstein, Cynthia Collings, David Main, Ghayyoor Ahmad, Sarah Naughton, Jayasimha Daddam, Vengai Mavangira, Mike Vandehaar, Zheng Zhou

**Affiliations:** 1 Department of Animal Science, Michigan State University, East Lansing, 48824 USA; 2 Department of Veterinary Diagnostic and Production Animal Medicine, Ames, 50011 USA

**Keywords:** Branched-chain amino acids, Branched-chain ketoacids, Fatty liver, Fresh cow

## Abstract

**Background:**

Dairy cows are at high risk of fatty liver disease in early lactation, but current preventative measures are not always effective. Cows with fatty liver have lower circulating branched-chain amino acid (BCAA) concentrations whereas cows with high circulating BCAA levels have low liver triglyceride (TG). Our objective was to determine the impact of BCAA and their corresponding ketoacids (branched-chain ketoacids, BCKA) on production performance and liver TG accumulation in Holstein cows in the first 3 weeks postpartum.

**Methods:**

Thirty-six multiparous Holstein cows were used in a randomized block design experiment. Cows were abomasally infused for the first 21 d postpartum with solutions of 1) saline (CON, *n* = 12); 2) BCA (67 g valine, 50 g leucine, and 34 g isoleucine, *n* = 12); and 3) BCK (77 g 2-ketovaline calcium salt, 57 g 2-ketoleucine calcium salt, and 39 g 2-ketoisoleucine calcium salt, *n* = 12). All cows received the same diet. Treatment effects were determined using PROC GLIMMIX in SAS.

**Results:**

No differences were detected for body weight, body condition score, or dry matter intake averaged over the first 21 d postpartum. Cows receiving BCK had significantly lower liver TG concentrations compared to CON (6.60% vs. 4.77%, standard error of the mean (SEM) 0.49) during the first 3 weeks of lactation. Infusion of BCA increased milk yield (39.5 vs. 35.3 kg/d, SEM 1.8), milk fat yield (2.10 vs. 1.69 kg/d, SEM 0.08), and lactose yield (2.11 vs. 1.67 kg/d, SEM 0.07) compared with CON. Compared to CON, cows receiving BCA had lower plasma glucose (55.0 vs. 59.2 mg/dL, SEM 0.86) but higher β-hydroxybutyrate (9.17 vs. 6.00 mg/dL, SEM 0.80).

**Conclusions:**

Overall, BCAA supplementation in this study improved milk production, whereas BCKA supplementation reduced TG accumulation in the liver of fresh cows.

**Graphical Abstract:**

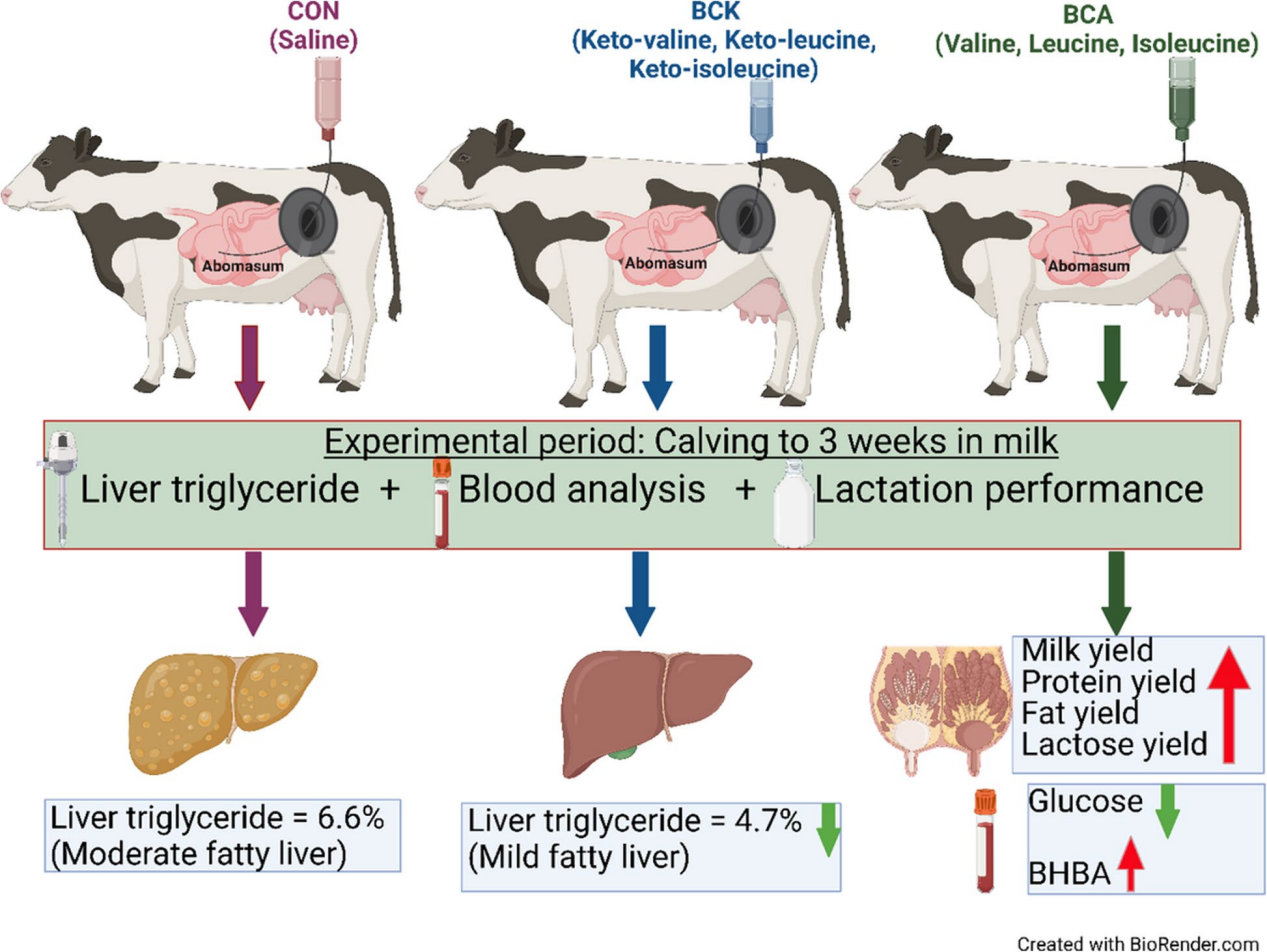

## Introduction

The annual cost of fatty liver disease (FL) in dairy cows in the United States was estimated to be over $150 million 8 years ago when prevalence was assumed at 4.8% [1]. FL in dairy cows is marked by accumulation of triglyceride (TG) within hepatocytes. Higher liver TG concentration is usually associated with compromised liver function and lactation performance [1, 2]. Current FL treatments are ineffective, as affected cows respond poorly to therapy with high mortality rates [3]. To alleviate the high rates of FL, many nutritional preventative measures have been developed for fresh cows [4]. Unfortunately, these strategies are not always effective [5–7].

Cows with FL have lower circulating branched-chain amino acid (BCAA) concentrations whereas cows with high circulating BCAA levels have low liver TG [1]. This inverse relationship has been well-established [1], but BCAA supplementation has not been tested as a strategy to reduce liver TG in fresh cows. As major components of milk protein and precursors for nonessential AA [8], only a small portion of BCAA in lactating cows is directly removed by the liver [9, 10]. In sheep, BCAA also had the lowest proportional extractions by the liver compared to other AA [11]. Although BCAA may not substantially impact the liver (as little BCAA are taken up by cow liver), transamination products of BCAA—branched-chain ketoacids (BCKA)—are catabolized in the liver in most mammals [12]. To our knowledge, uptake of BCKA by liver has not been shown in cattle, but in sheep, as much as 75% of BCKA are catabolized in the liver [13]. Hence, BCKA shuttled to the liver may serve as mediators of the inverse relationship between circulating BCAA and liver TG concentration and BCKA supplementation might suppress accumulation of liver TG in early postpartum cows. Therefore, increasing BCKA availability to the liver, either by post-ruminal BCKA supplementation or by enhancing BCAA transamination by post-ruminal BCAA supplementation may reduce liver TG concentration in fresh cows.

Recent work in early-lactation dairy cows has also demonstrated that BCAA supplementation improved milk yield and milk components yield [14]. Increased post-ruminal BCAA supply likely contributes to these responses, as BCAA are estimated to make up 20.8% of total AA mass in milk [15]. Although little is known about the impact of BCKA on lactation performance, feeding keto-leucine increased milk fat yield in cows during mid-lactation [16]. Therefore, our working hypothesis was that post-ruminal BCKA supplementation would reduce the risk of FL whereas post-ruminal BCAA supplementation would improve lactation performance in fresh cows. To that end, the objectives of this study were to evaluate the effect of abomasal BCAA and BCKA infusion on liver TG concentrations and lactation performance in fresh cows.

## Materials and methods

### Experimental design and dietary treatments

**Fig. 1:**
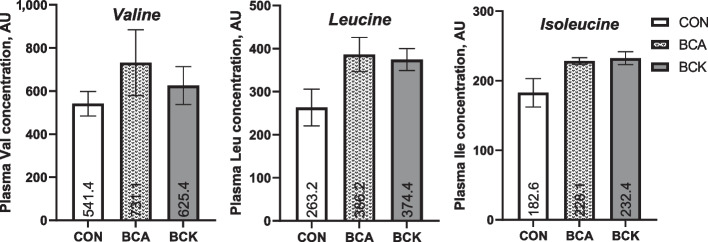
Plasma valine, leucine, and isoleucine concentrations in a preliminary block of 6 cows (*n* = 2/treatment) on d 3 postpartum

All procedures were approved by the Michigan State University Institutional Animal Care and Use Committee (protocol No. PROTO202000206). The experiment was conducted as a randomized complete block design with multiparous (lactation ≥ 2) Holstein dairy cows (*n* = 36) from the Michigan State University Dairy Cattle Teaching and Research Center (Lansing, MI, USA) between November 2021 and March 2022. A total of 36 cows were blocked according to expected calving date into 3 blocks. On average, cows enrolled had a 305-d mature equivalent milk yield of 13,421 ± 266 kg from previous lactation. Before enrollment, cows had an average body weight (BW) of 709 ± 8 kg and an average body condition score (BCS) of 3.3 ± 0.2. All cows underwent a rumenotomy procedure at 46.1 ± 2.2 d before expected calving date unless previously cannulated (*n* = 6). The number of animals included in this study were based on a power analysis to provide an 80% chance of detecting a 1.5% difference in liver TG concentration. Within each block, cows were randomly assigned to 1 of 3 treatments: control (CON, *n* = 12) receiving daily abomasal saline infusion without branched-chain amino acids or ketoacids; branched-chain amino acid (BCA, *n* = 12) receiving daily abomasal infusion of 67 g L-valine, 50 g L-leucine, and 34 g L-isoleucine (> 99.9% purity, ACP Chemicals, Montreal, QB, Canada); and branched-chain ketoacid (BCK, *n* = 12) receiving daily abomasal infusion of 77 g 2-ketovaline calcium salt, 57 g 2-ketoleucine calcium salt, and 39 g 2-ketoisoleucine calcium salt (> 99% purity, Stru Chem, Wujiang City, China). Using data from a previous study [17], plasma BCAA concentrations were on average 33% greater in cows with low hepatic TG (average 7.8 mg/g) whereas cows with high hepatic TG (average 50.8 mg/g) had lower plasma BCAA concentrations on d 4 postpartum. Therefore, dosage of BCA and BCK was based on a preliminary block of 6 cows (Fig. 1) that numerically increased plasma BCAA concentration by approximately 30%. The dose of BCA and corresponding BCK were matched on a 1:1 molar ratio across BCA and BCK treatments. Calcium chloride dihydrate (> 98% purity, Fisher Chemical) was added to CON and BCA infusate to match the calcium level in the BCK treatment. Treatments were delivered to cows daily via continuous abomasal infusion using a peristaltic pump [18, 19]. Seven liters of aqueous BCA (0.57 mol valine, 0.38 mol leucine, 0.26 mol isoleucine, pH = 2.5) or BCK (0.57 mol 2-ketovaline calcium salt, 0.38 mol 2-ketoleucine calcium salt, 0.26 mol 2-ketoisoleucine calcium salt, pH = 2.5) solution were administered to each cow daily. Calving day was considered d 0. Initiation of abomasal infusion for all animals occurred at 1200 h on d 1 after calving. Abomasal infusions were halted during milking for approximately 3–4 h/d. Abomasal infusion lines were flushed with 120 mL water after halting and before continuing infusion to ensure treatment delivery to abomasum.

### Animal management

Animals were housed in individual sawdust-bedded tie stalls and were milked 3 times per day at approximately 0630, 1430, and 2230 h. Animals were fed ad libitum at 115% of expected intake. All cows received a common lactation diet (Table 1) once daily in the morning from calving to 21 d in milk (DIM). All cows enrolled in the study received the same close-up diet prior to calving. The lactation diet was formulated in AMTS Farm Cattle (Agricultural Modeling and Training Systems, LLC; Groton, NY, USA) to meet nutrient requirements for lactating cows at 14 DIM with an expected milk yield of 36.3 kg/d with 3.7% fat and 3.0% protein.

**Table 1:** Ingredient and nutrient composition of the experimental diet fed to multiparous Holstein fresh cows

Item (DM basis)	Diet
Ingredient, %	
Corn silage	34.8
Ground corn	18.5
Alfalfa silage	15.4
Soybean meal	10.2
Whole cottonseed	8.43
Alfalfa hay	5.62
Bypass protein^a^	2.44
Mineral and vitamin premix^b^	1.97
Calcium carbonate	1.19
Protein blend^c^	1.14
Sodium sesquicarbonate	1.07
Urea	0.16
Rumen protected methionine^d^	0.07
Tallow	0.01
Nutrient, mean ± SD^e^	
CP, %	17.5 ± 0.5
aNDFom^f^	30.6 ± 0.8
ADF, %	20.9 ± 0.9
Lignin, %	3.6 ± 0.2
Starch, %	25.7 ± 1.3
Ether extract, %	4.3 ± 0.4
Ash, %	7.7 ± 1.1
Ca, %	1.17 ± 0.10
P, %	0.43 ± 0.02
Mg, %	0.30 ± 0.02
K, %	1.30 ± 0.07
Na, %	0.44 ± 0.03

^a^AminoPlus, Ag Processing Inc., Omaha, NE, USA

^b^The premix contained 30.5% Na, 7,567 mg/kg Zn, 3,888 mg/kg Mn, 961 mg/kg Cu, 105 mg/kg Co, 125 mg/kg I, 67 mg/kg Cr, 74 mg/kg Se, 1,930 IU/g vitamin A, 1,110 IU/g vitamin D_3_, and 29.66 IU/g vitamin E

^c^Caledonia Pass, Caledonia Farmers Elevator Co.; 73% CP, a mix of mostly porcine blood meal, feather meal, and grain byproducts

^d^Smartamine M, Adisseo, Alpharetta, GA, USA

^e^Means reported from composited weekly TMR samples (Cumberland Valley Analytical Services, Waynesboro, PA, USA)

^f^Amylase-treated NDF

Body weight was measured weekly for each cow at the same time before the morning feeding. A body condition score (BCS; scale 1 = thin to 5 = obese, with quarter-point increments) was assigned to each cow weekly by 3 individuals and the average score was used for statistical analysis [20]. Dry matter intake was recorded daily. Milk yield was recorded daily during the first 21 DIM. Milk composition was analyzed, whereas energy-corrected milk (ECM) and fat-corrected milk (FCM) were calculated from calving to 21 DIM.

General cow housing and healthcare conform to approved standard operating procedures for the Michigan State University Dairy Cattle Teaching & Research Center. Attending veterinarians from the Michigan State University conducted diagnosis and, when needed, performed treatment for health problems.

### Feed and milk sample collection and analyses

Dry matter (DM) of individual feed ingredients was determined weekly, and lactation diet was adjusted accordingly to maintain DM ratios of ingredients in the total mixed ration (TMR). Weekly samples of ingredients and TMR were frozen at –20 °C and composited monthly for analysis of DM, crude protein (CP), neutral detergent fiber (NDF), acid detergent fiber (ADF), amino acid (AA), lignin, ether extract, ash, Ca, P, Mg, K, Na by standard wet chemistry techniques [21] at a commercial laboratory (Cumberland Valley Analytical Services, Waynesboro, PA, USA) (Table 1). The values for net energy of lactation (NE_L_), rumen undegradable protein (RUP), rumen degradable protein (RDP), metabolizable protein (MP), Lys:Met ratio were also predicted using the National Academies of Sciences, Engineering, and Medicine (NASEM) 2021 model [15] with AA concentrations in the diet, dry matter intake (DMI), BW, BCS, milk production, and milk composition for each treatment as inputs (Table 2). Dry matter intake as a % of BW (DMI, % BW) was calculated daily using the weekly BW for each individual cow. Consecutive morning, midday, and evening milk samples were taken twice weekly. Milk samples were stored in a sealed tube with preservative (Bronopol tablet; D&F Control Systems, San Ramon, CA, USA) and stored at 4 °C until analyzed. Infrared spectroscopy was used to analyze and report milk fat (%), true protein (%), lactose (%), solids (%), and milk urea nitrogen (MUN) (mg/dL) and flow cytometry used to analyze somatic cell count (SCC) (1,000/mL) (Central Star Cooperative, Inc., Grand Ledge, MI, USA). Daily milk yield was summed over 3 milkings. Yields of 3.5% FCM and ECM were calculated as follows: FCM = 0.4324 × milk yield (kg) + 16.216 × fat yield (kg), ECM = 12.82 × fat yield (kg) + 7.13 × protein yield (kg) + 0.323 × milk yield (kg) [22, 23].

**Table 2:** Nutrient composition evaluation (NASEM 2021) of early lactation diet fed to multiparous Holstein fresh cows^a^

Component^c^	Treatments^b^		
	CON	BCA	BCK
NE_L_, Mcal/kg of DM	1.76	1.75	1.77
CP, % of DM	17.6	18.3	17.6
RDP, % of DM	12.0	11.9	12.0
NE_L_ allowable milk, kg/d	35.2	37.6 (35.7)	37.5
MP allowable milk, kg/d	29.1	34.0 (31.7)	29.8
Predicted milk protein, kg/d	0.87	0.88	0.93
Predicted milk, kg/d	36.9	37.4	38.6
MP supplied, kg/d	1.98	2.12	2.10
MP from Microbial CP, kg/d	1.11	1.12	1.17
MP from RUP, kg/d	0.87	1.00	0.93
MP balance, g/d	–333	–278	–292
Absorbed Val, g/d	121	186	129
Absorbed Leu, g/d	176	224	187
Absorbed Ile, g/d	118	147	125

^a^Predicted by Nutrient Requirements of Dairy Cattle (v.8, The National Academies of Sciences, Engineering, and Medicine) [15] based on ingredient composition, with the observed mean DMI, BW, BW loss, milk yield, and milk components for each treatment

^b^*CON* Control (saline 0.9%), *BCA* Branched chain amino acids (67 g valine, 50 g leucine, and 34 g isoleucine), *BCK* Branched chain keto-acids (77 g keto-valine, 57 g keto-leucine, and 39 g keto-isoleucine)

^c^Values were predicted using the NASEM Dairy software V8 R2022.12.08 using actual milk production and diets for each treatment. BCAA were entered as RUP supplements with 100% digestibility. BCKA were entered as a FA supplement with 100% digestibility to provide the same DE supply as BCKA. For BCA treatment, values for NE and MP-allowable milk are dependent upon the cow description. Values in parentheses were predictions using the milk production of CON cows to predict milk without the complicating effects of user-entered milk on urinary N output

### Blood sample collection and analysis

Blood was sampled from the coccygeal vein on 0 (actual calving day before treatment), 3, 7, 14, and 21 d relative to parturition before the morning feeding. Samples were collected into evacuated tubes (BD Vacutainer^®^, Becton, Dickinson and Company, Franklin Lakes, NJ, USA) containing either clot activator or sodium heparin for serum and plasma, respectively. After blood collection, tubes with sodium heparin were placed on ice and tubes with clot activator were kept at 21 °C until centrifugation (~ 30 min). Serum and plasma were obtained by centrifugation at 2,000 × *g* for 30 min at 4 °C. Aliquots of serum and plasma were frozen (–20 °C) until further analysis.

Commercial kits were used to analyze concentrations of plasma glucose (Sigma Chemical Co., St. Louis, MO, USA; Catalog number: P7119), serum insulin (Mercodia AB, Uppsala, Sweden; Catalog number 10-1201-01), plasma non-esterified fatty acid (NEFA, NEFA HR(2) kit, Wako Chemicals USA, Richmond, VA, USA), and plasma β-hydroxybutyrate (BHBA, Stanbio Laboratory, Boerne, TX, USA; Catalog number 2440-058) as described previously [24, 25]. All samples were analyzed in triplicate. Insulin resistance indices were calculated according to the following formulas [26]: homeostasis model assessment of insulin resistance (HOMA) = glucose (mmol/L) × insulin (µU/mL); quantitative insulin sensitivity check index (QUICKI) = 1/{(log[glucose (mg/dL)] + log[insulin (µU/mL)]}; and revised QUICKI (RQUICKI) = 1/{log[glucose (mg/dL)] + log[insulin (µU/mL)] + log[NEFA (mmol/L)]}.

### Liver sample collection and TG concentration quantification

Liver was sampled via puncture biopsy using a Hughes trocar over the 11^th^ and 12^th^ ribs [25] on d 1, 7, 14, and 21 relative to parturition. On biopsy days, animals were escorted from morning milking to animal handling chutes before having access to feed. Location of biopsy was determined by ultrasound. Biopsy incision site was at least 2 inches away from the previous incision site. An area of approximately 10 cm × 15 cm around the incision site was anesthetized with 10 mL of 2% lidocaine (Vet One, Boise, ID, USA). Biopsy samples were harvested and immediately frozen and stored in liquid nitrogen until further analysis for concentration of TG. Approximately 50 mg of liver was used for liver TG quantification in triplicates using the method previously described [25] except a five-point TG standard was created with a commercially available TG standard solution (Pointe Scientific, Canton, MI, USA; Catalog number: T7531STD).

### Statistical analysis

All data were analyzed using PROC GLIMMIX of SAS v9.4 (SAS Institute, Cary, NC, USA) with the model: *Y = µ + b*_*i*_*+ T*_*j*_*+ D*_*k*_*+ TD*_*jk*_*+ A*_*l:ij*_*+ е*_*ijklm*_ where *Y* = the dependent, continuous variable, *µ* = overall mean, *b*_*i*_*=* random effect of the *i*th block, *T*_*i*_ = fixed effect of treatment, *D*_*k*_ = fixed effect of time, *A*_*l:ij*_ = random effect of the *l*^th^ cow nested within block × Treatment and *е*_*ijklm*_*=* the residual error. Fixed effect of time is the day or week relative to parturition in which samples were collected for each variable. Parity (second vs. third lactation and greater) was kept in the model as covariate for all variables when significant (*P* ≤ 0.05). The blood and liver samples collected prior to administration of treatments (d 0 samples) were also included as covariate for NEFA, BHB, and liver TG concentration respectively if found to be significant. Blood metabolites and liver TG were analyzed at various time points that were not equally spaced with a heterogeneous variance over time. Therefore, the first order ante-dependence covariance structure ANTE(1), spatial power covariance structure SP(POW), or Toeplitz covariance structure (TOEP) was chosen based on Akaike’s information criterion values for repeated measures of liver TG and blood metabolites. For equally spaced data, autoregressive covariance structure AR(1) was used for repeated measures. Somatic cell count was log transformed for statistical analysis and presentation. Tukey’s post hoc test was used for multiple comparison correction of *P*-values for all pairwise comparisons of least squares means (LSM). Statistical differences were declared significant at *P* ≤ 0.05 and tendencies at 0.05 < *P* ≤ 0.10.

## Results

### Health

Health-related problems that occurred during the experiment are summarized in Table 3. Two cows (1 receiving BCA and 1 receiving BCK) developed peritonitis unrelated to experimental treatments (according to autopsy reports) and were removed from the study following the recommendation of attending veterinarians from the Michigan State University department of Large Animal Clinical Sciences. All data from these two cows were removed from the analysis. Five cows developed hypocalcemia; however, 4 of the 5 cases occurred before treatment (d 0 or d 1 relative to parturition). One cow from BCK had hypocalcemia on d 3 relative to parturition. One hypocalcemia cow from BCA also became ketotic on d 6 postpartum. The other 3 ketotic cows had moderate or high ketones during the second week of lactation.

**Table 3:** Frequency of health incidence in multiparous Holstein fresh cows

Variable	Treatments^a^		
	CON	BCA	BCK
Cows^b^	12	12	12
Ketosis^c^	1	2	1
Hypocalcemia^d^	2	2	1
Metritis	0	0	0
Displaced abomasum	0	0	0
Mastitis	0	0	0

^a^*CON* Control (saline 0.9%), *BCA* Branched chain amino acids (67 g valine, 50 g leucine, and 34 g isoleucine), *BCK* Branched chain keto-acids (77 g keto-valine, 57 g keto-leucine, and 39 g keto-isoleucine)

^b^Actual number of cows completing the study

^c^Defined as cows having moderate (~ 40 mg/dL) or large ketone concentrations (> 80 mg/dL) in urine and treated by veterinarians with oral propylene glycol

^d^Defined by recumbency and muscle tremors and received oral bolus of Bovikalc (Boehringer Ingelheim, USA)

### Dry matter intake, body weight, and body condition score

Dry matter intake, DMI%BW, BW, BW change, and BCS are presented in Table 4. As expected, DMI of cows in all treatments gradually increased (*P* < 0.01) from calving to 21 d in milk. However, DMI did not differ between treatment groups (*P* = 0.22). When normalized to BW (DMI %BW), intake also did not differ between treatment groups (*P* = 0.18). Similarly, BW, BCS, and BCS change were not affected by either treatment (*P* > 0.58) during the experimental period.

**Table 4:** Lactation performance in multiparous Holstein fresh cows

Parameter	Treatments^1^			SEM	*P*-value		
	CON	BCA	BCK		Trt	Time	Trt × Time
DMI, kg/d	18.9	19.2	20.4	0.61	0.22	< 0.01	0.40
DMI, %BW	2.95	2.90	3.11	0.12	0.18	< 0.01	0.42
BW, kg	644	644	660	13.0	0.58	< 0.01	0.95
BW change, kg/week	–15.6	–17.0	–14.4	3.4	0.86	0.03	0.88
BCS	3.16	3.12	3.12	0.08	0.92	< 0.01	0.73
Milk composition, %							
Fat	4.93	4.90	4.84	0.13	0.85	< 0.01	0.87
Protein	3.73^AB^	3.51^B^	3.83^A^	0.08	0.01	< 0.01	0.85
Lactose	4.88	4.86	4.83	0.02	0.28	< 0.01	0.26
Solids not fat or protein	5.74	5.70	5.70	0.03	0.51	< 0.01	0.11
MUN^2^, mg/dL	11.5^b^	12.7^A,a^	11.2^B^	0.40	0.02	0.11	0.78
SCC^3^	1.55	1.52	1.64	0.07	0.46	< 0.01	0.42
Milk production, kg/d							
Milk yield	35.3^B^	39.5^A^	35.1^B^	1.80	0.02	< 0.01	0.34
Fat yield	1.69^B^	2.10^A^	1.67^B^	0.08	0.02	0.02	0.72
Protein yield	1.25^b^	1.46^a^	1.30^b^	0.07	0.10	0.17	0.80
Lactose yield	1.67^B^	2.11^A^	1.69^B^	0.07	0.02	< 0.01	0.49
FCM^3^	41.1^B^	52.0^A,a^	42.0^b^	3.3	0.04	< 0.01	0.50
ECM^4^	40.8^b^	50.8^a^	41.8^b^	3.1	0.05	< 0.01	0.53
Milk:DMI	1.90^ab^	2.11^a^	1.79^b^	0.08	0.03	< 0.01	0.23

^A,B^Within a row, means with different capitalized superscripts differ after controlling for pairwise LSM comparisons using Tukey’s post-hoc procedure (*P* ≤ 0.05)

^a,b^Within a row, means with different superscripts (lowercase) tend to differ after controlling for pairwise LSM comparisons using Tukey’s post-hoc procedure (0.05 < *P* ≤ 0.10)

^1^*CON* Control (saline 0.9%), *BCA* Branched chain amino acids (67 g valine, 50 g leucine, and 34 g isoleucine), *BCK* Branched chain keto-acids (77 g keto-valine, 57 g keto-leucine, and 39 g keto-isoleucine)

^2^*MUN* Milk urea nitrogen

^3^Fat corrected milk calculated as FCM (kg) = 0.4324 × Milk yield (kg) + 16.216 × Fat yield (kg) [22, 23]

^4^Energy corrected milk calculated as ECM (kg) = 12.82 × fat yield (kg) + 7.13 × protein yield (kg) + 0.323 × milk yield (kg) [22, 23]

### Milk production and composition

**Fig. 2:**
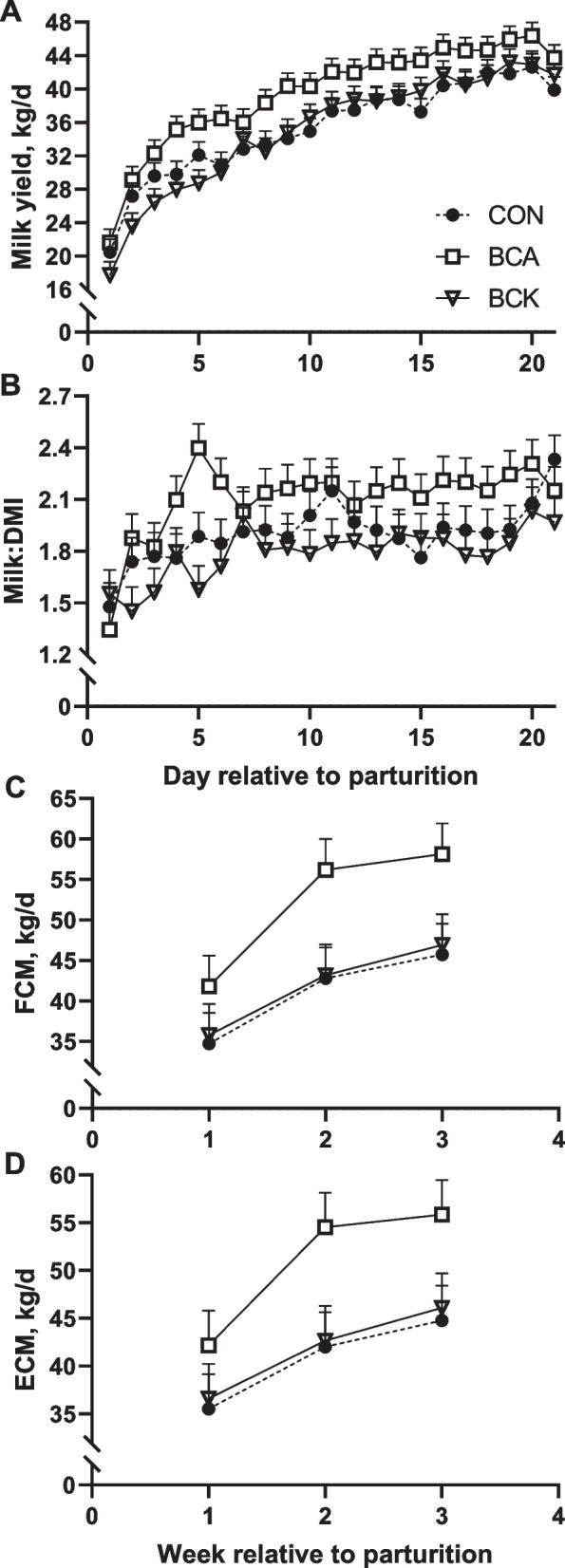
Effects of BCAA or BCKA infusion during early lactation on milk yield (**A**), milk:DMI (**B**), fat corrected milk (**C**) and energy corrected milk (**D**)

**Fig. 3:**
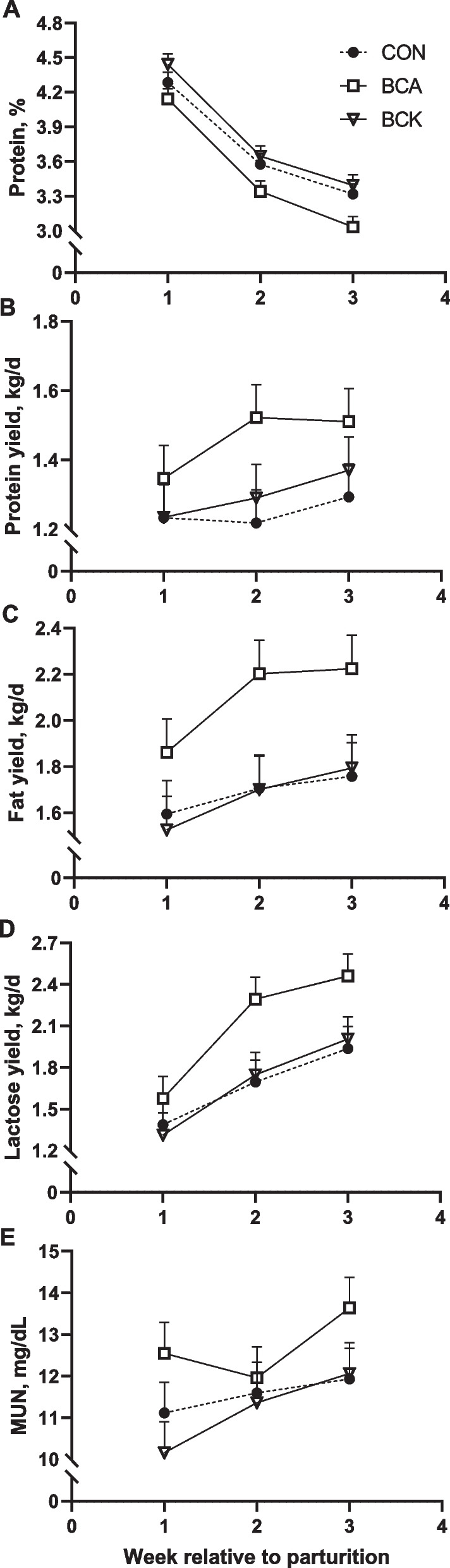
Effects of BCAA or BCKA infusion during early lactation on milk protein percentage (**A**), milk protein yield (**B**), milk fat yield (**C**), milk lactose yield (**D**) and milk MUN (**E**)

Milk production and composition variables are presented in Table 4. Overall dietary treatment effect was observed for milk yield, milk fat yield, lactose yield, ECM, FCM, milk:DMI, milk protein percentage, and MUN (*P* < 0.05). Specifically, BCA significantly increased milk yield compared to CON (*P* = 0.05) and BCK (*P* = 0.04) (Fig. 2A). Although milk fat and lactose percentage were not impacted by BCA or BCK treatments (*P* > 0.28), BCA significantly increased milk fat yield and milk lactose yield (*P* ≤ 0.05, Fig. 3D) as well as led to a tendency towards higher milk protein yield (*P* = 0.10) due to higher milk yield in BCA cows. Similarly, although milk protein percentage was significantly lower in BCA than BCK cows (*P* = 0.01, Fig. 3A), BCA cows had similar milk protein yield as BCK cows. The significantly higher milk:DMI in BCA cows (Fig. 2B) was also mainly driven by greater milk yield from BCA cows. As shown in Fig. 2C, FCM for cows receiving BCAA infusion was greater compared to CON (*P* = 0.05) and tended to be higher compared to BCK (*P* = 0.08). A tendency towards higher ECM (Fig. 2D) was also observed in BCA cows when compared to CON (*P* = 0.06) and BCK (*P* = 0.10). BCA significantly increased MUN compared to BCK (*P* = 0.02) whereas it led to only a tendency towards lower MUN when compared to CON cows (*P* = 0.10). Milk fat, lactose, and solids percentage as well as SCC were not affected by treatments (*P* ≥ 0.28).

### Blood biomarkers and liver TG concentration

**Fig. 4:**
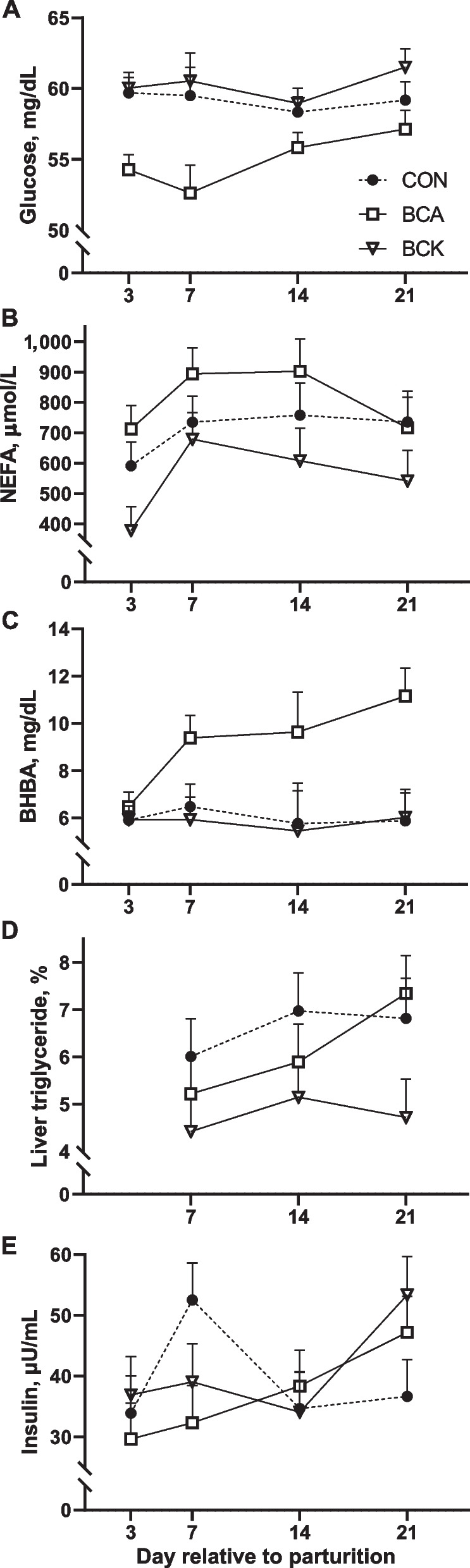
Effects of BCAA or BCKA infusion during early lactation on plasma concentrations of glucose (**A**), NEFA (**B**), BHBA (**C**), liver triglyceride (**D**), and insulin (**E**)

Blood glucose, insulin, NEFA, BHBA, and liver TG concentration are presented in Table 5. Overall treatment effect was observed for plasma glucose, BHBA, NEFA, and liver triglyceride concentration (*P* < 0.05). Specifically, cows receiving BCAA had significantly lower (*P* < 0.01) plasma glucose compared to CON and BCK cows. In contrast, plasma BHBA level in BCA cows were significantly higher than CON (*P* = 0.02) and BCK cows (*P* = 0.01). Although neither BCA nor BCK altered plasma NEFA concentrations compared to CON, plasma NEFA concentrations in BCA cows were higher (*P* = 0.02) than in BCK cows (Fig. 4B). Insulin and insulin resistance indices were not altered by BCA or BCK (*P* > 0.20). Compared to CON, BCK infusion significantly decreased liver TG (*P* = 0.03). In contrast, liver TG concentration in BCA cows tended to be higher (*P* = 0.09) compared to BCK cows (Fig. 4D).

**Table 5:** Blood and liver biomarker concentrations for multiparous Holstein fresh cows

Parameters	Treatments^1^			SEM	*P*-value		
	CON	BCA	BCK		Trt	Day	Trt × Day
Glucose, mg/dL	59.2^A^	55.0^B^	60.3^A^	0.86	< 0.01	0.29	0.58
Insulin, µU/mL	6.57	6.15	6.81	0.61	0.73	0.04	0.15
BHBA, mg/dL	6.00^B^	9.17^A^	5.83^B^	0.80	0.01	0.06	0.11
NEFA^2^, µmol/L	0.71^AB^	0.81^A^	0.55^B^	0.07	0.03	< 0.01	0.76
Liver triglycerides^3^, %	6.60^A^	6.15^AB,a^	4.77^B,b^	0.49	0.03	0.28	0.74
Insulin resistance index^4^							
HOMA	21.97	19.92	23.37	2.22	0.54	0.03	0.16
QUICKI	0.40	0.42	0.41	0.01	0.20	0.05	0.16
RQUICKI	0.44	0.45	0.45	0.01	0.36	< 0.01	0.25

^A,B^Within a row, means with different capitalized superscripts differ after controlling for pairwise LSM comparisons using Tukey’s post-hoc procedure (*P* ≤ 0.05)

^a,b^Within a row, means with different superscripts (lowercase) tend to differ after controlling for pairwise LSM comparisons using Tukey’s post-hoc procedure (0.05 < *P* ≤ 0.10)

^1^*CON* Control (saline 0.9%), *BCA* Branched chain amino acids (67 g valine, 50 g leucine, and 34 g isoleucine), *BCK* Branched chain keto-acids (77 g keto-valine, 57 g keto-leucine, and 39 g keto-isoleucine)

^2^Nonesterified fatty acids

^3^Liver triglycerides reported as % wet tissue weight

^4^HOMA (homeostasis model assessment) = glucose (mmol/L) × insulin (µU/mL); QUICKI (quantitative insulin-sensitivity check index) = 1/{log[glucose (mg/dL)] + log[insulin (µU/mL)]}; RQUICKI (revised QUICKI) = 1/{log[glucose (mg/dL)] + log[insulin (µU/mL)] + log[fatty acids (mmol/L)} [26]

## Discussion

### Milk production and composition

Abomasal infusion of BCAA at the current dose increased milk yield by 4.2 kg/d compared to CON. Based on NASEM [15] predictions for our diets and assuming that 100% of infused AA were absorbed (Table 2), cows infused with BCAA were predicted to produce 2.5 kg more milk per day based on protein supply if no change was made in user-entered milk yield. It is well-established that milk yield is highly correlated with mammary plasma flow [27]. A recent jugular infusion study demonstrated that Leu (50 g/d) and Ile (22 g/d) supplementation increased mammary plasma flow by 24% and increased milk yield by 2.3 kg/d [14]. In the current study, we infused Leu at the same dose as Yoder et al. [14] and Ile at a higher dose (34 g/d) into the abomasum. Hence, the increased milk yield of BCA cows in this study might have been partly caused by a higher mammary plasma flow. Measurement of mammary plasma flow will be required to confirm this speculation.

Abomasal infusion of BCAA at the current dose also increased milk protein yield by 0.21 kg/d compared to CON. This outcome was expected considering fresh cows are in negative MP balance [28], and increasing the amount of AA available may alleviate MP shortage in dairy cows by providing more metabolizable AA available as precursors for milk protein. However, measurement of mammary AA uptake will be required to confirm this speculation. We suggest that supplementing BCAA may also have stimulated milk protein synthesis and milk yield via activation of intracellular signaling pathways, such as the mammalian target of rapamycin (mTOR) pathway, based on work in other species and in cultured mammary cells and tissue [29–31]. As a coordinator of cell growth and metabolism, mTOR signaling plays a central role in regulating many fundamental cell processes, especially protein synthesis [32]. Therefore, we expected BCAA supplementation to upregulate milk protein synthesis. However, the effect of BCAA supplementation on lactation performance in published literature has been inconsistent. Yoder et al. [14] found that milk yield increased in response to jugular infusion of Leu (50 g/d) and Ile (22 g/d) and speculated that this increase was likely due to changes in mammalian target of rapamycin complex 1 (mTORC1). In contrast, Leal Yepes et al. [33] observed no increase in milk yield with supplementation of rumen-protected BCAA (61 g/d Val, 101 g/d Leu, and 41 g/d Ile) during the first 35 d in milk. Such inconsistencies of BCAA effect on milk production could be due to differences in BCAA dosage and method of delivery between these experiments. In the present study, we infused BCAA via continuous abomasal infusion using the molar ratio (Val:Leu:Ile = 2.2:1.5:1) observed in plasma of fresh cows with low liver TG concentration from a previous transition cow study [17]. Because BCAA-infused cows produced 4.2 kg/d more milk than CON, with little change in milk protein and fat percentage in milk, they also produced more fat, and protein. In contrast to the effects of BCAA, infusion of BCKA at equal molar amounts and ratio did not alter milk production. The only previous report on BCKA infusion was a study demonstrating that dietary supplementation of keto-leucine at 0.75% of DM in a partially rumen-protected form increased milk fat yield in dairy cows [16]. It is possible that the results of that study were due to similarities between ketoleucine and isovalerate, one of the branched-chain VFAs required by cellulolytic bacteria in the rumen [34].

The milk:DMI ratio is an estimate of gross feed efficiency, although this ratio does not account for BW change in individual cows. With BW change similar between groups, milk:DMI provides a good estimate of feed efficiency in this study. Therefore, BCA cows were more efficient than CON cows, with a 4.2 kg/d increase in milk yield without increased DMI.

### Glucose, insulin, and insulin resistance indices

It has been well established that a low-level of insulin could induce a metabolic condition favoring tissue and AA catabolism in cows, which might result in lactose yield increase because catabolism of gluconeogenic AA could increase the glucose available for mammary gland to produce lactose [35]. Although only a numerical decrease in insulin was observed in response to BCAA supplementation in this study, the observed increase in lactose yield may partially originate from increased AA catabolism. However, future evidence quantifying changes in gluconeogenesis in response to BCAA or BCKA supplementation may be required to support this hypothesis. Previous reports from both monogastric and ruminant studies have also illustrated the impact of BCAA supplementation on circulating glucose concentration [36, 37]. As the main precursor for lactose production in the mammary gland, mammary glucose uptake greatly impacts the circulating glucose concentration in dairy cows, especially during early lactation when cows enter a phase of negative energy balance [38, 39]. In this study, BCAA infusion decreased plasma glucose concentration, but significantly increased lactose yield compared with CON. A recent report in dairy cows revealed that Leu and Ile supplementation increased mammary plasma flow [14]. Although increase in mammary plasma flow could increase mammary glucose uptake, which, in turn, might have caused the lower plasma glucose concentration observed in this study, measurement of mammary arteriovenous glucose concentration difference will be required to confirm this speculation.

Recent advances in human research suggest that increases in BCAA plasma levels are associated with insulin resistance, and that elevated BCAA may cause insulin resistance by stimulating mTORC1 [30]. Contradictory to the suggested causal relationship, recent work in humans and rodents suggest that increased circulating BCAA concentrations are simply markers of insulin resistance, as physiological changes in BCAA concentrations are not sufficient to elicit insulin resistance [40, 41]. In dairy cows, homeorhetic adaptations to ensure sufficient glucose supply for the mammary gland induce insulin resistance in extra-mammary tissues during early lactation [42]. In this study, no change in insulin or various insulin resistance indices (HOMA, QUICKI, and RQUICKI) were observed in response to BCA or BCK, which could be because blood samples were collected after a short fasting period (~ 3 h). Hence, intravenous glucose and/or insulin tolerance test after overnight fasting may be required to determine the casual relationship between BCAA supplementation and insulin resistance in fresh cows.

### Liver triglyceride concentration, BHBA, and NEFA

Fatty liver in dairy cattle is categorized into mild (1%–5% TG wet weight), moderate (5%–10% TG wet weight), and severe FL (> 10% TG wet weight) [1]. In the present study, BCK cows were in the mild FL category with an average 4.77% liver TG concentration whereas CON and BCA cows were in the moderate FL category with average liver TG concentration of 6.60% and 6.15%, respectively. Previous report from a murine model demonstrated that BCAA supplementation decreased liver TG [43]. Similarly, in dairy cattle, supplementation with BCAA and propylene glycol also reduced liver TG in early lactation [33]. With 40%–50% of dairy cows having liver TG concentration > 5% within the first month after calving, FL in dairy cows is usually associated with compromised lactation performance and increased susceptibility to health issues [1]. Based on these associations, strategies that effectively reduce liver TG concentration are expected to improve lactation performance in dairy cows by decreasing negative impact of FL disease. Despite the significantly lower liver TG concentration in BCK cows, lactation performance was not improved in the first 3 weeks of lactation. In contrast, with liver TG concentration in the moderate FL category, BCA increased milk yield and milk components yield compared to CON cows in the first 3 weeks of lactation. These results suggest that liver TG concentration may not be the best predictor for lactation performance, at least in fresh cows with liver TG concentration in the mild or moderate FL category. Instead, alterations in liver TG may reflect the degree of adipose tissue mobilization and consequent elevation in circulating NEFA concentrations in fresh cows. However, quantifiable measurements of adipose tissue mobilization will be required to confirm this speculation.

It is also noteworthy that abomasal infusion of BCAA at current dose did not decrease liver TG compared to CON, but infusing equal molar amount of BCKA (deaminated BCAA) successfully decreased liver TG concentration. This is likely because after absorption, BCAA were minimally removed by the liver in dairy cows whereas BCKA absorbed were metabolized primarily in the liver [44, 45]. Additionally, a proportion of BCAA was also likely utilized for milk and tissue protein synthesis during early lactation, which likely contributes to the discrepancy of BCA and BCK impact on liver TG concentration. Apart from the amount of BCKA reaching the liver, liver TG concentration is also greatly impacted by circulating NEFA concentrations during early lactation, as circulating NEFA concentration peaks soon after parturition in dairy cows [46]. Although circulating NEFA concentrations in BCA cows and BCK cows were similar to CON cows, BCA cows had significantly higher NEFA concentrations compared to BCK cows. Considering hepatic uptake of NEFAs is impacted by concentration of NEFAs in blood in cows [47], the significantly higher circulating NEFA level in BCA cows was likely associated with the observed tendency towards higher liver TG concentration in BCA cows compared to cows infused with BCK.

As a marker for adipose tissue lipolysis, very high levels of circulating NEFA concentrations in early lactation are associated with compromised health in dairy cows [48]. Nevertheless, NEFA are also important sources of energy and fatty acids for liver and mammary gland [28]. Therefore, a moderate increase in circulating NEFA concentration that provides appropriate amount of energy substrate without jeopardizing health outcome may be beneficial for fresh cows. Although circulating NEFA concentration was not altered in response to rumen-protected BCAA supplementation at a different dose in a recent fresh cow study [49], NEFA concentration in BCA cows was significantly higher than BCK cows and numerically higher than CON cows in this study. Meanwhile, milk fat yield in BCA cows was significantly higher than CON and BCK cows. Taken together, these results suggest that a higher circulating NEFA in BCA cows from this study could contribute to increased milk fat yield. However, data on mammary NEFA uptake will be required to confirm this speculation.

The early lactation period in dairy cows is typically marked with elevated plasma BHBA due to tissue mobilization [50]. Excessive circulating BHBA (subclinical or clinical ketosis) is typically associated with decreased milk production and potential health consequences, such as increased incidence of hyperketonemia and FL disease and decreasing β-oxidation, TCA cycle activity, and gluconeogenesis in hepatocytes [1, 51–53]. Although a previous report did not observe any change in circulating BHBA concentrations in response to BCAA supplementation [33], plasma BHBA concentrations were significantly higher in BCA cows compared with CON and BCK cows in this study. It is important to note that BHBA concentrations in the BCA cows of this study averaged 9.17 mg/dL (0.88 mmol/L). Therefore, these cows were not in a state of subclinical (1.2–2.9 mmol/L) or clinical (> 3.0 mmol/L) ketosis [54]. Considering BHBA is a main substrate for milk fat synthesis in the mammary gland [51], elevated circulating BHBA levels below the threshold for subclinical ketosis (1.2 mmol/L) may also contribute to the higher milk fat yield in BCA cows by providing more substrate for de novo FA synthesis in the mammary gland in this study. However, more direct evidence (e.g., mammary BHBA uptake or de novo milk FA output) will be required to support this speculation. As a main product of ketogenesis in the liver, higher circulating BHBA in BCA cows could also be an indicator of elevated incomplete FA oxidation in the liver. However, measurement of changes in abundance and activities of enzymes controlling ketogenesis are required to confirm such speculation.

## Conclusions

With liver TG concentration in CON cows in the moderate FL category and TG concentration in BCK cows in the mild FL category, lower liver TG concentrations in BCK cows compared to CON cows suggests that post-ruminal BCKA supplementation at the current dose may be an effective measure for minimizing risk of moderate or severe FL in cows during early lactation. Although abomasal BCAA infusion did not decrease liver TG concentration, the greater milk yield and components yield compared to CON cows suggest that post-ruminal BCAA supplementation may improve lactation performance in fresh cows. The discrepancy in lactation performance and liver TG concentration between BCA and BCK cows in this study were likely due to differences in metabolic alterations in response to BCAA and BCKA, as BCA cows had significantly lower circulating glucose, higher NEFA, and higher BHBA levels compared to BCK cows. Considering these divergent responses were attained from same molar amounts of post-ruminal supplementation of BCAA and BCKA in this study, future research should focus on the interrelated metabolism of BCAA and BCKA to better understand the unique effects on lactation performance and underlying metabolic mechanisms.

## Data Availability

The datasets used and/or analyzed during the current study are available from the corresponding author on reasonable request.

## Abbreviations

AA: Amino acid
ADF: Acid detergent fiber
BCAA: Branched-chain amino acid
BCA: BCAA-infused group
BCKA: Branched-chain ketoacids
BCK: BCKA-infused group
BCS: Body condition score
BHBA: β-hydroxybutyrate
BW: Body weight
CON: Control group
CP: Crude protein
DIM: Days in milk
DM: Dry matter
DMI: Dry matter intake
ECM: Energy-corrected milk
FCM: Fat-corrected milk
FL: Fatty liver
GLIMMIX: Generalized linear mixed model
HOMA: Homeostasis model assessment
MP: Metabolizable protein
MUN: Milk urea nitrogen
mTOR: Mammalian target of rapamycin
mTORC1: Mammalian target of rapamycin complex 1
NASEM: National Academies of Sciences, Engineering, and Medicine
NEFA: Non-esterified fatty acid
NDF: Neutral detergent fiber
NE_L_: Net energy of lactation
QUICKI: Quantitative insulin sensitivity check index
RQUICKI: Revised QUICKI
RUP: Rumen undegradable protein
RDP: Rumen degradable protein
SCC: Somatic cell count
SEM: Standard error of the mean
TG: Triglyceride
TMR: Total mixed ration
